# Organizational Context Matters: Interdepartmental Collaboration and Performance in Law Enforcement vs. Scientific Research Agencies

**DOI:** 10.3390/bs16050787

**Published:** 2026-05-15

**Authors:** Hyung-Woo Lee, Dong-Young Rhee

**Affiliations:** 1 Department of Public Administration, Hannam University, Daejeon 34430, Republic of Korea; hwlee@hnu.kr; 2 Department of Politics and Public Administration, Hallym University, Chuncheon 24252, Republic of Korea

**Keywords:** collaboration, law enforcement, scientific research agency, performance improvement

## Abstract

This study challenges the assumption that collaboration beyond work-unit boundaries consistently enhances performance and demonstrates that the impacts of inter-unit collaboration are not uniform across different government agencies. Using multi-group analysis of Federal Employee Viewpoint Survey (FEVS) data, we compare two U.S. federal agencies with contrasting institutional logics: a bureaucratic law enforcement agency (ICE) and an entrepreneurial science research center (GSFC). In doing so, we propose a model explaining how inter-unit collaboration influences performance through knowledge acquisition and resource availability as mediating variables. The analysis revealed that inter-unit collaboration had a significantly greater impact on work unit performance in the bureaucratic ICE than in the entrepreneurial GSFC. Specifically, the total effect of inter-unit collaboration on unit performance was 0.166 for ICE and 0.087 for GSFC, whereas the total effect of intra-unit collaboration reached 0.525 and 0.686, respectively. These findings contradict our hypothesis that inter-unit collaboration will be more beneficial in entrepreneurial agencies. Looking closely at the mediating paths, we argue that high technical complexity and rigid task compartmentalization in scientific agencies create barriers to lateral sharing, whereas law enforcement tasks are often case-based and emergency-driven and require more effective real-time coordination among units. Paradoxically, this unexpected result reinforces our initial assumption that inter-unit collaboration can produce diverse outcomes by showing that the nature of tasks and the resulting collaboration structures—factors we initially overlooked—serve as contingencies for performance gains.

## 1. Introduction

Collaboration has emerged as a defining feature of contemporary public administration, supported by multiple streams of research that changed its role in modern governance (Ansell & Gash, 2008; Emerson & Nabatchi, 2015). The underlying rationale for collaboration varies depending on its level. At the interorganizational level, collaboration is primarily motivated by the pursuit of democratic ideals and inclusive governance. Recent studies emphasize that collaborative governance fosters legitimacy and accountability by engaging diverse stakeholders across sectors (Bjärstig et al., 2024; Pulido-Gómez et al., 2025). While an economic rationale—such as reducing costs through resource pooling from private-sector engagement (Elston et al., 2018)—remains relevant, the central objective is to incorporate multiple perspectives, thereby enhancing demographic responsiveness and public value (Page et al., 2015). At the intra-organizational level, on the contrary, extending the scope of collaboration beyond the traditional boundaries of work units is primarily undertaken with the goal of enhancing performance. The New Public Management (NPM) movement has advocated reforms modeled on private-sector practices to remedy government inefficiency. Early reformers, notably Osborne and Gaebler (1992), argued that customer orientation should take precedence over procedural accountability. From this perspective, public organizations are encouraged to promote inter-unit collaboration as a means of improving customer satisfaction.

Yet this broad endorsement of inter-unit collaboration is often underpinned by an implicit assumption: that public organizations are structurally and functionally uniform. In reality, the public sector comprises a diverse constellation of agencies with varying missions, technical demands, and professional cultures. Law enforcement units, scientific research institutions, regulatory commissions, and social service providers each operate under distinct task environments and accountability frameworks (Bozeman & Bretschneider, 1994; Romzek & Dubnick, 1987). Treating these disparate organizations as a homogenous category risks oversimplifying policy prescriptions and misaligning collaborative strategies.

Importantly, collaboration is not uniformly enacted. Inter-unit collaboration often hinges on the discretion of managers (Park et al., 2021), whose perceptions and incentives play a pivotal role (Kanon, 2024). If managers lack motivation or view collaboration as inconsequential to their own unit’s performance, efforts may remain superficial or unfulfilled. The anticipated value of collaborative initiatives is highly contingent on organizational context. For example, bureaucratic organizations characterized by centralized authority, formal rules, and risk aversion may struggle with cross-functional collaboration due to procedural rigidity and compartmentalized roles (O’Toole et al., 2005; Kelling & Moore, 1989). In contrast, entrepreneurial public organizations—such as scientific research agencies—often depend on inter-unit collaboration to synthesize diverse expertise and adapt to uncertainty (Gustetic et al., 2015; Bugos & Boyd, 2008). Accordingly, applying collaborative prescriptions indiscriminately, without regard for structural and functional variation, may therefore lead to limited or even counterproductive outcomes.

This study addresses this gap by comparing collaboration dynamic s across two types of public organizations with distinct institutional logics: a bureaucratic law enforcement agency and an entrepreneurial scientific research agency. Specifically, we examine whether inter-unit collaboration enhances work unit performance through two mediating mechanisms, knowledge availability and resource acquisition, and whether these relationships differ between ICE and GSFC. By doing so, this study makes three contributions. First, it challenges the assumption that inter-unit collaboration universally improves performance in public organizations. Second, it identifies the mediating mechanisms through which collaboration affects performance. Third, it demonstrates that the performance value of collaboration depends not only on organizational culture but also on task characteristics, specialization, and resource transferability.

In the theory section, we first develop a mediating model to explain the performance outcomes of inter-unit collaboration. The model incorporates two mediating paths, reflecting resource acquisition and knowledge improvement as the major theoretical perspectives. We also include the effect of intra-unit collaboration as a baseline for comparison with inter-unit collaboration. Second, we advance a hypothesis regarding the moderating effect, positing that bureaucratic versus entrepreneurial culture acts as a contingency for performance outcomes.

## 2. Theory and Hypotheses

### 2.1. How Does Collaboration Improves Performance?

The compartmentalized model of organization has increasingly been challenged. Functional silos assume that goals can be decomposed into discrete, independent tasks and delegated accordingly. Yet, modern organizations—particularly those in dynamic public sectors—face complex and interdependent problems that demand integrated solutions. As a result, cross-functional (inter-unit) collaboration has gained increasing importance across sectors (Ahmad et al., 2023).

Two theoretical perspectives underscore this shift. First, knowledge-based theories of organization emphasize that organizational knowledge is the most strategically significant resource for improving performance (Argote & Ingram, 2000; Grant, 1996; Wiig, 2002). Cross-functional collaboration facilitates the combination of specialized knowledge from distinct units, allowing for novel insights and more adaptive solutions (Argote & Ingram, 2000). For example, when technical, operational, and administrative units share expertise, the organization’s collective problem-solving capacity increases (Tsai, 2002). This trend is further emphasized in the context of digital government (Ahmad et al., 2023; Keppler & Leonardi, 2023). Empirical studies in both public and private settings confirm that interdepartmental knowledge integration supports innovation and service quality (Lin, 2007; Love & Roper, 2009).

The growing importance of transactive memory systems (Wegner, 1987) in organization studies further illustrates how inter-unit collaboration enhances organizational performance. In highly collaborative environments, employees do not merely possess domain-specific knowledge; they also know who within the organization knows what, making it easier to locate and apply relevant expertise across units (Moreland, 1999; Lewis, 2004). This mechanism reduces redundant efforts and accelerates coordinated action. Cross-functional collaboration facilitates knowledge sharing by breaking down bureaucratic rigidities (M. B. O’Leary et al., 2011). In particular, organizations operating in uncertain or turbulent environments benefit more from horizontal coordination than from vertical control (Lawrence & Lorsch, 1967; Uhl-Bien et al., 2007). Taken together, these perspectives suggest that cross-functional collaboration is not merely a coordination tool, but a strategic capability that enhances the organizational knowledge base and, ultimately, improves performance.

**Hypothesis** **1.** *Inter-unit collaboration enhances knowledge availability, thereby improving work unit performance.*

Second, resource dependence theory posits that no organizational unit operates in complete isolation from its environment. Rather, departments and subunits are embedded in a web of interdependencies, wherein they must interact with others to secure critical resources such as personnel, equipment, and funding (Hillman et al., 2009). In complex bureaucracies, especially those with matrix or networked structures, cross-departmental collaboration becomes a strategic necessity (Ford & Randolph, 1992; Hite & Hesterly, 2001).

Cross-functional collaboration enhances resource sufficiency in several ways. First, it facilitates pooled access to scarce resources, especially when budgets are tight or resource allocation is highly politicized. Second, it reduces duplication of efforts and promotes complementarity between departments, allowing each unit to leverage the strengths and inputs of others. Third, in environments where resources are distributed unevenly, collaboration allows less-resourced units to gain access to specialized tools, staff, or infrastructure that they would otherwise lack. Research has shown that collaboration beyond boundaries allows for more efficient use of administrative capacity and can offset personnel shortages in mission-critical areas (Larkin et al., 2011).

This logic is particularly relevant in the public sector, where agencies increasingly employ cross-functional teams, joint task forces, and one-stop service models to overcome functional fragmentation and resource silos (Liu & Zheng, 2018; Ryu & Rainey, 2009). Taken together, the interdependence of departmental resource needs and the benefits of collaborative resource sharing suggest that inter-unit collaboration can significantly improve a department’s resource sufficiency, and in turn, its performance.

**Hypothesis** **2.** *Inter-unit collaboration improves resource acquisition, thereby enhancing work unit performance.*

**Figure 1:**
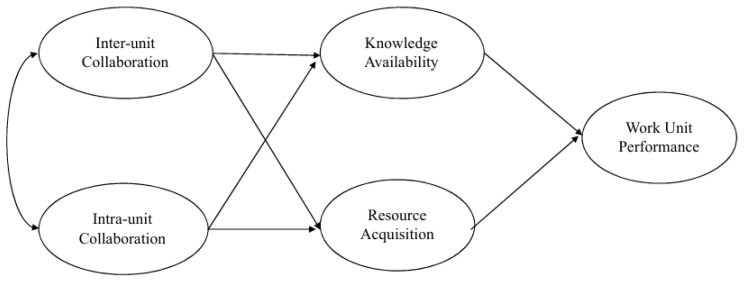
Conceptual Model.

Building on these hypotheses, the study develops the research framework shown in Figure 1. We added the independent variable of intra-unit collaboration in the model against which the effects of inter-unit collaboration are to be compared.

### 2.2. The Diversity of Public Organizations

Traditionally, public administration scholars have often treated public organizations as a relatively homogeneous category, primarily defined by their governmental funding and legal mandates. However, this assumption has increasingly come under scrutiny. Bozeman and Bretschneider (1994) challenged the rigid public–private dichotomy by introducing the concept of “publicness,” which views public organizations as heterogeneous entities varying in ownership, funding, political influence, and task environments. Similarly, Romzek and Dubnick (1987) emphasized that even within the public sector, agencies operate under divergent professional norms, accountability structures, and operational logics—factors that lead to distinctive organizational behavior. Drawing on contingency theory (Lawrence & Lorsch, 1967), one can argue that different environmental demands shape varying internal structures and collaboration strategies.

#### 2.2.1. Bureaucratic Orientation and Intra-Unit Collaboration

A key dimension of variation among public organizations is bureaucratic intensity—the degree to which agencies rely on formal rules, hierarchy, and standardized procedures to govern operations. Although public agencies generally exhibit higher levels of bureaucracy than private firms, the degree of bureaucratization differs across domains. Law enforcement agencies, for example, tend to prioritize procedural justice, impartiality, and risk mitigation. These goals are best achieved through centralized authority, strict adherence to protocol, and internal discipline (Kelling & Moore, 1989; Nicholson-Crotty & O’Toole, 2004). Bureaucratic structures in such agencies reinforce vertical accountability and may discourage discretion and innovation (Maynard-Moody & Musheno, 2003).

As a result, collaboration across departments in highly bureaucratic environments may be constrained by jurisdictional boundaries, information silos, and task segmentation. In contrast, collaboration within units—among employees who share training, task objectives, and reporting lines—is often more feasible and productive. Intra-unit collaboration under bureaucratic systems tends to be rule-bounded yet effective in improving performance because it operates within a clearly defined procedural framework (O’Toole et al., 2005). Research in police departments shows that internal cohesion and unit-level coordination correlate with both efficiency and public trust (de Carvalho et al., 2023).

**Hypothesis** **3.** *In organizations with strong bureaucratic orientation, intra-unit collaboration has a greater impact on work unit performance.*

#### 2.2.2. Entrepreneurial Orientation and Inter-Unit Collaboration

In contrast to bureaucratic rigidity, the New Public Management (NPM) movement—influenced by private sector managerialism—has encouraged public agencies to adopt more entrepreneurial practices. These include decentralized authority, performance-based accountability, and innovation-oriented leadership (Osborne & Gaebler, 1992). The degree of NPM adoption varies across agencies; while some public agencies have embraced horizontal governance structures and flexible teamwork (Stewart & Kimber, 1996), others have retained their traditional, hierarchical frameworks (Parker & Bradley, 2000).

Scientific research agencies—such as NASA, NOAA, or national labs—exemplify this entrepreneurial orientation within the public sector. Their primary missions involve solving ill-structured problems, pursuing innovation, and generating new knowledge—tasks that necessitate cross-functional collaboration, multidisciplinary integration, and adaptive learning (Bugos & Boyd, 2008; Heracleous & Gonzalez, 2014). These agencies are often project-based, where team composition cuts across traditional departmental lines, and knowledge exchange is facilitated through matrix structures or task force arrangements.

Prior studies of public R&D organizations have shown that inter-unit collaboration improves task flexibility and knowledge flow, which are crucial for managing scientific uncertainty (Bozeman & Boardman, 2003; Li & Tang, 2020). For example, NASA’s crowdsourced innovation initiatives and coordinated committees for technology development demonstrate how horizontal coordination can lead to breakthroughs that siloed teams could not achieve independently (Gustetic et al., 2015). In such contexts, performance is less about control and more about integration—how effectively knowledge, expertise, and resources are mobilized across boundaries.

**Hypothesis** **4.** *In organizations with strong entrepreneurial orientation, inter-unit collaboration has a greater impact on work unit performance.*

## 3. Methods

### 3.1. Data, Sample, and Analysis

This study draws on the Federal Employee Viewpoint Survey (FEVS) administered by the U.S. Office of Personnel Management (OPM), which has annually surveyed federal employees since 2010. This study primarily analyzed the 2019 FEVS data. While it would be ideal to select the most recent data from the 2025 survey, questions related to inter-unit collaboration have been omitted starting in 2020 due to the inclusion of items related to COVID-19. The FEVS includes items assessing work experiences, leadership, and organizational attitudes. In 2019, 1,443,152 employees were invited to participate, and 615,395 responded (42.6% response rate). Of the sample, 39% were from headquarters and 61% from field offices. A majority were non-supervisors (64%). 44% of the respondents were female; 65% identified as white, and 26% of the respondents served their agencies more than 20 years.

To evaluate the proposed model, the items related to the research variables were selected from the FEVS data. We conducted Structural Equation Modeling (SEM) using the sample from Immigration and Customs Enforcement (ICE) within the Department of Homeland Security, and Goddard Space Flight Center (GSFC) within the National Aeronautics and Space Administration. ICE was selected as a representative bureaucratic law enforcement agency because its mission involves rule enforcement, procedural compliance, and hierarchical accountability. GSFC was selected as our presentative entrepreneurial scientific research center because its mission involves scientific discovery, technological innovation, and project-based knowledge production. Respondents who failed to complete any relevant survey items were excluded, resulting in a final sample size of 6833 for ICE and 1719 for GSFC. To assess organizational differences in collaboration dynamics, we apply Multiple Group Analysis (MGA) for comparative evaluation across the two groups.

### 3.2. Measurement

To measure the two key independent variables—inter-unit collaboration and intra-unit collaboration—two FEVS 2019 items were selected for each. Inter-unit collaboration was assessed via the statements: “Managers promote communication among different work units (e.g., about projects, goals, needed resources) (Q58),” and “Managers support collaboration across work units to accomplish work objectives (Q59),” yielding a high internal consistency (Cronbach’s α = 0.948). Intra-unit collaboration was measured by: “The people I work with cooperate to get the job done (Q20),” and “Employees in my work unit share job knowledge with each other (Q26).” Although the first item lacks explicit reference to the “work unit,” its survey placement under the heading “My Work Unit” supports its contextual validity. Cronbach’s α for this construct was 0.770.

Two mediating variables are knowledge availability and resource acquisition. Knowledge availability was captured using: “My work unit has the job-relevant knowledge and skills to accomplish organizational goals (Q29).” Knowledge availability was measured with a single item because the FEVS contains only one item that directly captures whether a work unit possesses the job-relevant knowledge and skills required to accomplish organizational goals. Although single-item measures have limitations, this item is conceptually specific and closely aligned with the construct. Resource acquisition included: “I have sufficient resources (e.g., people, materials, budget) to get my job done (Q9),” and “I have enough information to do my job well (Q2)” (α = 0.689).

To align the analytical scope with the mechanisms of intra- and inter-unit collaboration, this study adopts work unit performance as the dependent variable rather than overall organizational performance. This decision reflects both theoretical and methodological considerations. Conceptually, collaborative behaviors—such as information sharing, resource exchange, and joint problem-solving—typically occur within or across functional units, and their direct effects are more immediately observable at the unit level. Additionally, constructs such as skill improvement and resource sufficiency, which mediate the relationship between collaboration and performance, are inherently tied to specific working unit or team contexts rather than the entire agency. Methodologically, the Federal Employee Viewpoint Survey (FEVS) items used in this study are based on employee perceptions of their immediate work environment and are framed around unit-level dynamics, making work unit performance a more valid and reliable outcome measure. Therefore, the use of work unit performance ensures coherence between theoretical constructs, measurement instruments, and the level of analysis employed. Work unit performance was measured by: “How would you rate the overall quality of work done by your work unit (Q28),” and “The skill level in my unit have improved in the past year (Q27)” (α = 0.707).

See Table 1 for construct items and reliability details.

### 3.3. Analysis Strategy

The main objective of this study is to demonstrate that inter-unit collaboration has varying impacts on performance across agencies. To this end, we propose a mediating model to examine the mechanisms through which collaboration influences performance and compare the results between two agencies: ICE and GSFC. To test the hypotheses presented in Figure 1, we proceed as follows. First, we conducted Structural Equation Modeling for each agency. Second, we compared the model paths between the two agencies.

## 4. Results

### 4.1. Preliminary Analysis

We assumed that ICE exhibits a strong bureaucratic characteristic, while GSFC demonstrates an entrepreneurial orientation. Although this proposition is grounded in theoretical foundations discussed earlier, its empirical validity requires verification prior to the main analysis. To assess this, we compared the mean responses to selected items from the FEVS 2019 that reflect degrees of bureaucratic and entrepreneurial culture.

The two concepts emphasize different foci. Organizations with a bureaucratic culture tend to stress the manner in which tasks are carried out (i.e., emphasis on hierarchy), whereas those with an entrepreneurial culture prioritize the achievement of improved outcomes (i.e., innovation) (Shelton & Lahtinen, 2025). Nevertheless, a significant correlation may exist between the two: bureaucratic culture acts as a hindrance to pursuing entrepreneurial orientation, while entrepreneurial reform requires dismantling bureaucratic barriers (Osborne & Gaebler, 1992).

First, two FEVS 2019 items capturing entrepreneurial culture were analyzed: “I feel encouraged to come up with a new and better way of doing things (Q3),” and “Creativity and innovations are rewarded (Q32).” Across all three items, GSFC reported higher mean values than ICE, with differences statistically significant according to *t*-tests.

Second, two items from the FEVS 2019 were selected to assess bureaucratic culture. Bureaucratic organizations are typically rule-bound, where individuals are given less discretion since centralized decision-making characterizes bureaucratic organizations. Two items—“Employees have a feeling of personal empowerment with respect to work processes (Q30)” and “How satisfied are you with your involvement in decisions that affect your work? (Q63)”—were used as indicators of bureaucratic culture after reverse coding.

ICE exhibited higher mean values on these bureaucratic measures and lower on entrepreneurial measures than GSFC, with statistically significant differences, supporting our classification. Table 2 summarizes these comparative results.

Given that all variables were measured using data from a single source, the analysis may be subject to common method bias. To assess this possibility, we conducted Harman’s one-factor test. First, all measurement items were loaded onto a single latent factor, and the resulting model fit was examined. The one-factor model exhibited poor fit (CMIN = 8652.583, SRMR = 0.1149, GFI = 0.744, NFI = 0.784, RFI = 0.698, IFI = 0.785, TLI = 0.698, CFI = 0.785, RMSEA = 0.225). In contrast, the five-factor model proposed in this study yielded good fit (CMIN = 816.672, SRMR = 0.0258, GFI = 0.980, NFI = 0.983, RFI = 0.966, IFI = 0.983, TLI = 0.966, CFI = 0.983, RMSEA = 0.096), thereby supporting the measurement validity of the constructs.

A measurement invariance test was conducted only using the latent variables. All chi-square difference tests indicated that CMIN changed significantly at each successive step. However, because chi-square difference tests are highly sensitive to sample size, and our sample exceeds several thousand cases, these results are less meaningful in practical terms. The unconstrained model demonstrated excellent fit indices (CFI = 0.989, SRMR = 0.0199), providing a strong baseline. The configural model also showed good fit (CFI = 0.987, RMSEA = 0.044), supporting configural invariance and indicating that the same factor structure holds across groups. When factor loadings were constrained to equality (metric model), model fit deteriorated substantially (χ^2^ = 1231.818, CFI = 0.968, ΔCFI = −0.019), suggesting that full metric invariance was supported only under more lenient criteria (Rutkowski & Svetina, 2014). Next, the loadings of Q2 and Q27 were freed across groups. The partial metric model exhibited improved fit (χ^2^ = 837.545, CFI = 0.979, ΔCFI = −0.008), meeting the recommended threshold (ΔCFI ≤ 0.01). This indicates that partial metric invariance was achieved, allowing for meaningful comparison of structural paths across groups. Table 3 presents measurement invariance test.

Table 4 and Table 5 present descriptive statistics and correlations among the variables. The mean values for all variables were higher in GSFC compared to ICE. In particular, the key independent variable—inter-unit collaboration—was significantly greater in GSFC (t = 29.33, *p* < 0.001). The correlations among the independent variables ranged from 0.437 to 0.630, all below the threshold of 0.70, indicating that multicollinearity is not a significant concern in this analysis.

### 4.2. Structural Equation Modeling

All fit indices for the structural model analysis, except for Chi-square, indicated a reasonable model fit: SRMR = 0.047, GFI = 0.943, NFI = 0.952, RFI = 0.918, IFI = 0.953, TLI = 0.919, CFI = 0.953, and RMSEA = 0.077. Although the Chi-square was significant (χ^2^ = 2161.79, *p* < 0.01), this is expected given its sensitivity to sample size (Zheng & Bentler, 2025). Thus, the overall model fit is deemed acceptable.

**Figure 2:**
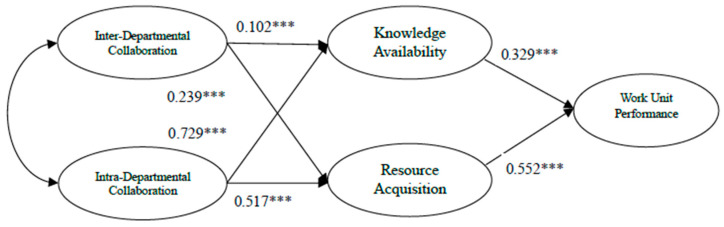
Structural Equation Model for ICE. *** significant paths.

**Figure 3:**
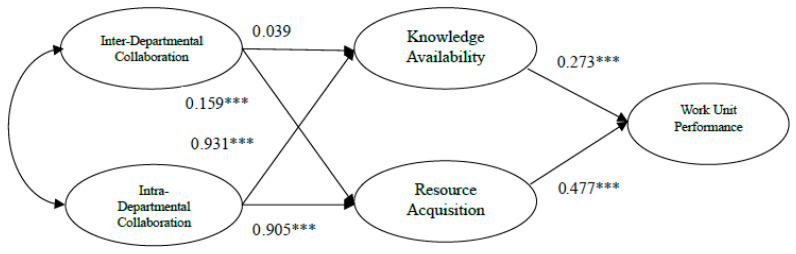
Structural Equation Model for GSFC. *** significant paths.

Figure 2 and Figure 3 present standardized path coefficients for ICE and GSFC. To aid readability, inter-construct covariances are omitted, though both independent variables showed significant correlations (0.653 for ICE, 0.341 for GSFC; *p* < 0.01). The result exhibits that intra-unit collaboration exerted greater influence than inter-unit collaboration in both agencies, with the disparity being more substantial in GSFC.

In the ICE model, all paths were significant, with the strongest effect between intra-unit collaboration and knowledge availability (0.729), and the weakest between inter-unit collaboration and knowledge availability (0.102). Inter-unit collaboration exerted a total effect of 0.166 on performance, while intra-unit collaboration showed a stronger total effect of 0.525.

In GSFC, all paths were significant except the links between inter-unit collaboration and knowledge availability (0.039); the strongest effect was found in the path from intra-unit collaboration to knowledge availability (0.931). Intra-unit collaboration played a more substantial role in driving performance than inter-unit collaboration. Specifically, the total effect of inter-unit collaboration on unit performance was 0.087, whereas the total effect of intra-unit collaboration reached 0.686. Examining individual paths reveals further insights. Resource acquisition was more heavily influenced by intra-unit (0.905) than inter-unit collaboration (0.159). Knowledge availability followed a similar trend: 0.931 for intra and 0.039 for inter-unit.

Table 6 summarizes the results of hypothesis testing. Hypothesis 1 was supported only in the case of ICE, whereas Hypothesis 2 received support in both agencies. Hypotheses 3 and 4 were not supported, as intra-unit collaboration exhibited a stronger total effect than inter-unit collaboration in both agencies. This pattern was more pronounced at GSFC, where reversed causalities were observed.

Table 7 summarizes the result of Multiple Group Analysis (MGA). Most path coefficients show significant difference between ICE and GSFC. Among all the path coefficients, ICE exhibited higher causal effects than GSFC only in three relationships: the impacts of inter-unit collaboration on knowledge availability and on resource acquisition, and the effect of knowledge availability on work-unit performance. For all other paths, GSFC showed stronger coefficients.

In both organizations, intra-unit collaboration played a more substantial role in driving performance than inter-unit collaboration. Notably, intra-unit collaboration exhibited a stronger impact on unit performance in GSFC compared to ICE. Specifically, the total effect of inter-unit collaboration on unit performance was 0.166 for ICE and 0.087 for GSFC, whereas the total effect of intra-unit collaboration reached 0.525 and 0.686, respectively.

Examining individual paths reveals further insights. In ICE, resource acquisition was influenced by intra-unit (0.517) and inter-unit collaboration (0.239). Knowledge availability was influenced by intra-unit (0.729) and inter-unit collaboration (0.102). Such disparity becomes pronounced for GSFC: resource acquisition shows a dominant influence from intra-unit collaboration (0.905) over inter-unit (0.159). Knowledge availability followed a similar trend: 0.931 from intra vs. 0.039 from inter-unit.

## 5. Conclusions

### 5.1. Implications 

This study examined the effects of inter-unit collaboration on unit performance by comparing two federal agencies with distinct missions and structural logics. A pivotal, yet counterintuitive, finding emerged: inter-unit collaboration exerted a more substantial impact on work unit performance in the bureaucratic agency (ICE) than in the entrepreneurial agency (GSFC), as indicated in the greater total effect of inter-unit collaboration at ICE (0.166 > 0.087). This contradicts the initial hypothesis that entrepreneurial organizations, characterized by innovation and flexibility, would be more dependent on cross-boundary collaboration for performance.

Several contextual factors elucidate this unexpected pattern. GSFC’s architecture is defined by high professionalization and extreme task specialization. Despite its entrepreneurial mission, GSFC is structured to solve well-defined technical problems where overarching goals are decomposed into compartmentalized tasks. In Raveendran et al. (2020) framework, goal interdependence at GSFC does not necessarily translate into task or knowledge interdependence. Our interpretation is also reinforced by the finding that the effect sizes of intra-unit collaboration on knowledge availability (0.931 > 0.729) and resource acquisition (0.905 > 0.517) were consistently greater at GSFC.

In this environment, information and material resources are often non-transferable because each unit operates within a highly distinct scientific domain. Hence, communication primarily follows vertical hierarchies rather than extending beyond horizontal boundaries, and middle managers—often technical experts rather than coordination specialists—may lack the capacity or incentive for consistent inter-unit integration. This aligns with prior research suggesting that elevated technical complexity hinders cross-unit compatibility, as functional misalignment often leads to integration challenges (Brusoni et al., 2001).

In contrast, ICE is designed to cope with emergency situations that are less programmable and require real-time, ad hoc inter-unit coordination. ICE’s mission execution begins when a case arises. Because cases often involve emergency situations, multiple departments are mobilized to dedicate their resources to address them. Since such emergency cases involve considerable uncertainty, formal top-down structures are often insufficient, highlighting the importance of middle managers in facilitating informal information flows across boundaries.

The nature of shared assets also plays a role: whereas GSFC shares specialized equipment that is hard to transfer, ICE primarily shares personnel, a resource that is more easily reallocated to meet immediate needs. GSFC mainly recruits personnel for each department based on specific scientific domains, whereas ICE tends to recruit generalists and provides opportunities to gain experience across various related units through internal job rotation. Thus, ICE is more likely to retain human capital essential to effective inter-unit collaboration.

In sum, these findings suggest that the efficacy of collaboration is not a simple function of mission orientation (entrepreneurial) or degree of autonomy (bureaucratic) but is shaped by a complex interplay between task characteristics, structural design, and the transferability of resources. The findings echo Galbraith’s (1973) suggestion that efforts to enhance organizational performance should take a holistic approach, in which restructuring is accompanied by the alignment of strategy, work processes, rewards, and personnel: simply institutionalizing inter-unit collaboration is insufficient. Also, this underscores the necessity for context-sensitive collaboration strategies that align with the complex realities of public agencies.

### 5.2. Discussions

This study challenges the assumption that collaborative practices uniformly enhance performance across public organizations. By comparing collaboration dynamics in two structurally distinct agencies—a bureaucratic law enforcement agency (ICE) and an entrepreneurial scientific research center (GSFC)—we demonstrate that the impact of intra- and inter-unit collaboration is highly context-dependent.

We initially expected inter-unit collaboration to have stronger performance effects in GSFC because scientific research agencies are commonly characterized as innovation-oriented, project-based, and dependent on multidisciplinary knowledge integration. Contrary to this expectation, the results showed that inter-unit collaboration had a weaker total effect in GSFC than in ICE. The contradicting result suggests that bureaucratic and entrepreneurial criteria represent only one dimension among many that define organizational characteristics. It appears that overlooked dimensions—the degree of specialization driven by the nature of tasks and collaboration structures—have played significant roles determining the success of inter-unit collaborations.

Notably, a *t*-test result reveals that although the managers at GSFC (the mean value of inter-unit collaboration was 4.02) demonstrated significantly greater proactiveness in fostering inter-unit collaborations compared to ICE (3.22), its impact was more pronounced in ICE. This indicates that despite of managerial efforts to encourage inter-unit collaboration, a range of factors may hinder the effectiveness of such efforts. This is particularly informative in the public sector context where agencies are under stronger institutional pressure for collaboration beyond departmental boundaries, since it implies that departmental leaders should make strategic choices that take into account their organization’s and department’s circumstances (R. O’Leary & Vij, 2012).

However, this unexpected result paradoxically strengthens our research thesis that inter-unit collaboration can yield diverse outcomes by illustrating that numerous factors may determine its impact. In particular, the nature of tasks and the collaboration structures arising from them—factors we initially overlooked—served as contingencies shaping performance gains from inter-unit collaboration. It also underscores the need for further empirical research to unpack the contingencies that moderate collaboration outcomes.

### 5.3. Limitations of the Study

This study is not entirely free from limitations. A key methodological concern relates to the measurement of latent constructs. In our SEM model, four latent variables were operationalized with only two observed indicators, and one variable with a single indicator. This limitation was largely unavoidable given the reliance on open-source survey data from FEVS. Although the model fit indices remained within acceptable ranges, the restricted number of items may constrain the reliability and validity of the constructs. Future research should incorporate a larger set of indicators to enhance measurement robustness and provide a more comprehensive representation of the underlying concepts.

In addition, the study may be subject to common method bias. Although Harman’s one-factor test suggested that the proposed five-factor model provided a substantially better fit to the data than a single-factor model, this does not eliminate the possibility of bias. Future research should explicitly address this concern by employing alternative research designs or statistical remedies.

On a related note, the dependent variable, work unit performance, was measured and analyzed at the individual level. In this sense, it should be more appropriately labelled as “perceived” work unit performance, and conclusions should be discussed with this distinction in mind. However, this study did not account for the discrepancy in the level of analysis. Although such an issue may represent a typical limitation of many empirical studies (Zhang et al., 2023), future research should address this concern in the design stage.

Future research also should broaden the analytical scope by including a wider array of moderating factors to examine how collaboration mechanisms interact with structural hybridity, leadership configurations, and digital infrastructures. Doing so will help refine collaboration models that reflect the realities of public sector diversity and advance performance in context-sensitive ways.

## Figures and Tables

**Table 1:** Variables and survey items.

Variables (Cronbach’s α)	Survey Items
Inter-unit Collaboration (α = 0.948)	“Managers promote communications among different work units (for example, about projects, goals, and needed resources).” “Managers support collaboration across work units to accomplish work objectives.”
Intra-unit Collaboration (α = 0.770)	“The people I work with cooperate to get the job done.” “Employees in my work unit share job knowledge with each other.”
Knowledge Availability	“My work unit has the job-relevant knowledge and skills necessary to accomplish organizational goals.”
Resource Acquisition (α = 0.689)	“I have sufficient resources (for example, people, materials, budget) to get my job done.” “I have enough information to do my job well.”
Work Unit Performance (α = 0.707)	“How would you rate the overall quality of work done by your work unit?” “The skill level in my work unit has improved in the past year.”

**Table 2:** Difference between ICE and GSFC.

Variable	Questionnaire Item Number	Groups	Mean	Mean Difference	t	*p*
Entrepreneurial Culture	Q3	ICE	3.34	−0.894	−32.07	0.000
		GSFC	4.24			
	Q32	ICE	2.94	−1.085	−38.30	0.000
		GSFC	4.02			
Bureaucratic Culture	Q30(R)	ICE	2.91	0.992	30.67	0.000
		GSFC	1.92			
	Q63(R)	ICE	2.67	0.771	24.18	0.000
		GSFC	1.90			

**Table 3:** Measurement Invariance Test.

Model	χ^2^ (CMIN)	df	SRMR	GFI	CFI	RMSEA
Unconstrained	468.77	14	0.0199	0.987	0.989	0.062
Configural	499.28	28	0.0210	0.986	0.987	0.044
Metric	1231.8	40	0.0457	0.969	0.968	0.059
Partial (Q2, Q27 freed)	837.55	36	0.0275	0.979	0.979	0.051

**Table 4:** Descriptive Statistics and Correlation Matrix for ICE.

	Mean (Std. Dev.)	Work Unit Performance	Inter-Unit Collaboration	Intra-Unit Collaboration	Resource Acquisition
Work Unit Performance	3.87 (0.90)				
Inter-unit Collaboration	3.22 (1.28)	0.557 ***			
Intra-unit Collaboration	3.89 (0.97)	0.700 ***	0.515 ***		
Knowledge Availability	3.97 (1.00)	0.698 ***	0.494 ***	0.598 ***	
Resource Acquisition	3.15 (0.1.07)	0.495 ***	0.571 ***	0.437 ***	0.486 ***

*** *p* < 0.01.

**Table 5:** Descriptive Statistics and Correlation Matrix for GSFC.

	Mean (Std. Dev.)	Work Unit Performance	Inter-Unit Collaboration	Intra-Unit Collaboration	Resource Acquisition
Work Unit Performance	4.25 (0.68)				
Inter-unit Collaboration	4.02 (0.93)	0.561 ***			
Intra-unit Collaboration	4.31 (0.71)	0.668 ***	0.556 ***		
Knowledge Availability	4.41 (0.74)	0.675 ***	0.507 ***	0.630 ***	
Resource Acquisition	3.87 (0.87)	0.466 ***	0.519 ***	0.499 ***	0.498 ***

*** *p* < 0.01.

**Table 6:** The Results of Hypothesis Testing.

No	ICE	GSFC
H1: Inter-unit Collaboration → Knowledge Availability → Performance	Supported	Not supported
H2: Inter-unit Collaboration → Resource Acquisition → Performance	Supported	Supported
H3: Intra-unit Collaboration has a greater impact on performance in bureaucratic agencies. H4: Inter-unit Collaboration has a greater impact on performance in entrepreneurial agencies.	Reversed correlations observed	

**Table 7:** Path Coefficients and Multiple Group Analysis.

	Path Coefficients		Chi-Squares
	ICE	GSFC	
Inter-unit Collaboration → Knowledge Availability	0.102 **	0.039	4.74 *
Intra-unit Collaboration → Knowledge Availability	0.729 **	0.931 **	15.27 **
Inter-unit Collaboration → Resource Acquisition	0.239 **	0.159 **	6.06 *
Intra-unit Collaboration → Resource Acquisition	0.517 **	0.905 **	50.32 **
Resource Acquisition → Work-unit Performance	0.552 **	0.477 **	2.70
Knowledge Availability → Work-unit Performance	0.329 **	0.273 **	4.16 *

* *p* < 0.05; ** *p* < 0.01.

## Data Availability

No new data were created or analyzed in this study.
